# Participants’ Evolving Experiences, Hope, and Coping While Enrolled in a Community-Based Bereavement Support Program: A Pre–Post Mixed-Methods Pilot Study

**DOI:** 10.3390/curroncol33060350

**Published:** 2026-06-10

**Authors:** Yoojung Kim, Carmen G. Loiselle

**Affiliations:** 1 Division of Experimental Medicine, Faculty of Medicine and Health Sciences, McGill University, Montreal, QC H4A 3J1, Canada; 2 Gerald Bronfman Department of Oncology, Faculty of Medicine and Health Sciences, McGill University, Montreal, QC H4A 3T2, Canada; 3 Ingram School of Nursing, Faculty of Medicine and Health Sciences, McGill University, Montreal, QC H3A 2M7, Canada

**Keywords:** bereavement, cancer, community-based, support program, mixed-methods

## Abstract

*Living with Loss*, an eight-session community-based bereavement program, supports individuals who have lost a loved one to cancer within the past two years. This pilot study sought to document people’s experiences across program delivery and compare levels of hope and coping before and after program completion. Participants (*N* = 11) completed self-report e-questionnaires (Hope Herth Index and Brief Cope Scale) before and after program completion. Semi-structured individual interviews were conducted before program attendance, at the midpoint and after program completion. Levels of hope and coping were compared using paired-sample *t*-tests. Digitally recorded interview data were transcribed verbatim and thematically analyzed. Total hope scores significantly increased at program completion. There were no significant pre-post changes in coping subscales. Qualitative data revealed three themes: (1) tangible and intangible program contributions to bereavement processes (i.e., developing coping strategies, perspective changes), (2) an enhanced sense of community post-attendance (i.e., mutual understandings, group-related growth), and (3) distinct preferences for in-person vs. virtual delivery (i.e., depth of interpersonal connections, anticipated instrumental issues including parking restrictions). Preliminary findings suggest that *Living with Loss* is relevant and beneficial for bereaved participants. Documenting experiences across program delivery serves to further inform program adjustments to better meet participants’ needs and preferences.

## Simple Summary

Community-based bereavement programs play an integral supportive role for individuals affected by the death of a loved one from cancer or other serious illnesses. However, there is limited literature on how participants benefit from these programs. *Living with Loss* is one such community-based program designed to support bereaved individuals. To address this gap, a pilot study (*N* = 11) was conducted to document participants’ evolving experiences, as well as to compare hope and coping before and after program completion. Hope significantly increased after program completion compared to pre-program levels. Participant interviews revealed that the program was valued as a resource for learning new coping strategies, gaining diverse perspectives on loss, and sharing mutual understandings. Emotional and logistical challenges were reported to be significantly associated with in-person participation. Together, these findings identify both the value of support and areas for optimizing program delivery.

## 1. Introduction

Cancer remains a leading cause of death in Canada, and the number of cases and deaths continues to rise worldwide [1,2]. Its impact commonly extends beyond the diagnosis, deeply affecting family members who often serve as informal caregivers [3,4]. These caregivers often experience a wide range of burdens—physical, emotional, economic and social—which may vary across different stages of the cancer trajectory [4,5,6]. Challenges faced by caregivers can lead to significant distress, negatively impacting their overall quality of life and well-being [7]. Moreover, when facing the death of their loved one, the burden persists through grief as a deeply emotional response to loss and bereavement [8,9]. Being able to adjust over time is essential for healing; however, the needs of caregivers following a patient’s death are frequently unmet [10].

Community-based organizations play an increasingly crucial role in addressing the needs of individuals who are affected by cancer, highlighting the importance of the active participation of community partners within cancer care [11,12,13]. These organizations are essential for providing a wide range of supportive services [14]. One such organization is *Hope and Cope*, based in Montreal, Quebec, Canada [15]. Focused on supporting individuals impacted by cancer, *Hope and Cope* comprises healthcare professionals and over 300 trained volunteers. It offers a wide range of services, including peer support groups and wellness activities (i.e., cooking and exercise classes) aimed at providing mental, physical, practical and psychosocial support to patients and families across various stages of cancer, including bereavement [15].

Although community-based support programs fill important support gaps [14,16,17], documenting their impact remains imperative. This pilot study aims to document such an impact in the context of an eight-session biweekly bereavement program Facilitated by a healthcare professional (a social worker), the program is offered to individuals who lost a loved one to cancer within two years of program registration [18].

More specifically, this study seeks to: (1) explore participants’ experiences across program delivery and (2) compare levels of hope and coping before and after program attendance.

## 2. Materials and Methods

### 2.1. Sample, Design, Procedures

#### 2.1.1. Sample

Individuals (*N* = 11) who registered for the *Living with Loss* program were recruited using convenience sampling [19]. Inclusion criteria were: (1) being at least 18 years of age, (2) being able to communicate in English, (3) providing informed consent, and (4) being officially registered for the program.

#### 2.1.2. Design

**Figure 1:**
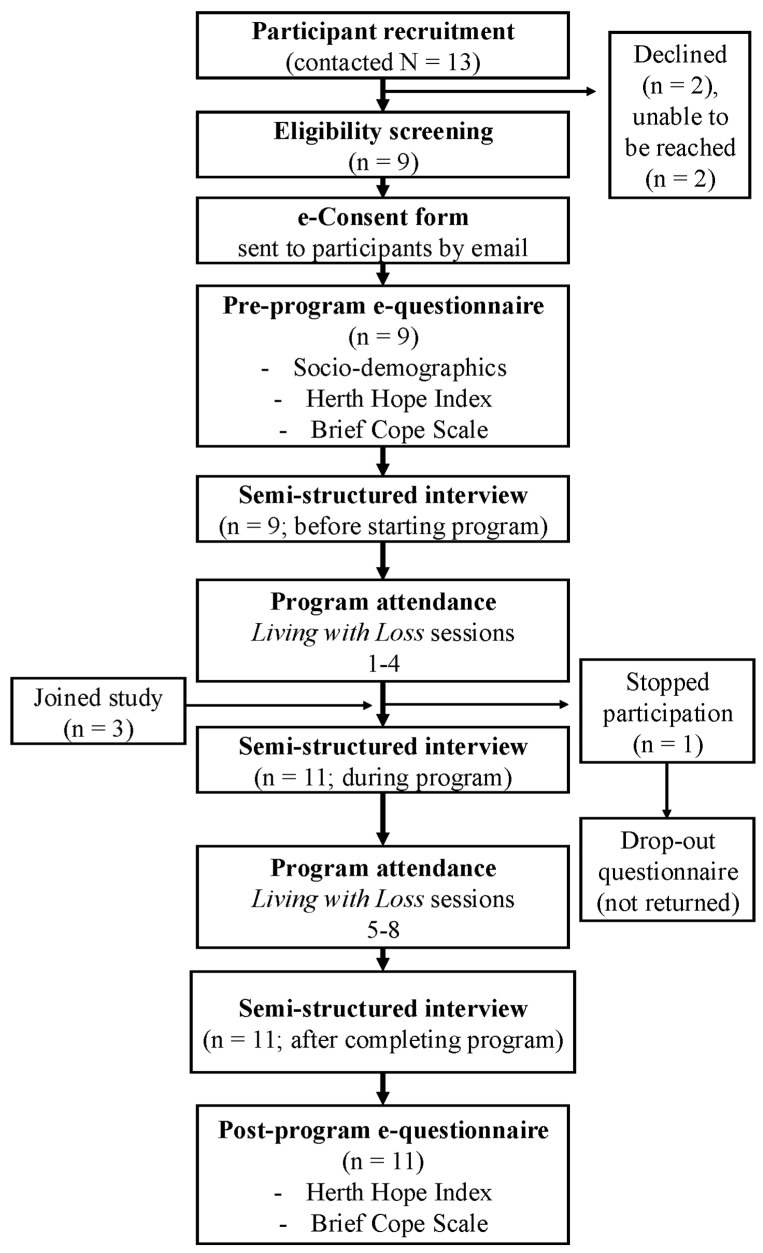
Study data collection flowchart.

Using a mixed-method design, self-report e-questionnaires were used to gather socio-demographic data and responses on validated instruments. Semi-structured interviews guided qualitative data collection before, during, and after program attendance (Figure 1).

#### 2.1.3. Procedures

Recruitment took place in two consecutive *Living with Loss* groups (conducted from May to July 2024 and from September to December 2024). Program delivery took place in-person at the Hope and Cope Wellness Center in Montreal, Quebec, Canada and was facilitated by the same healthcare professional (a social worker) for both groups. Table S1 provides content details.

Program registrants who consented to being contacted for research purposes were reached by phone by the first author (Y.K) and screened for eligibility. Those who were eligible and interested were provided with study details, and an e-consent form was sent electronically. Upon consenting, participants completed the pre-program study materials and a one-on-one interview: before the program began (T1), mid-program attendance (i.e., after 4 sessions, T2) and at program completion (T3). Participants were offered a $10 gift card for each e-questionnaire and interview completed (i.e., from either Best Buy, Amazon, or Starbucks).

### 2.2. Data Collection

#### 2.2.1. Self-Report E-Questionnaires

Socio-demographic data were collected at baseline. Hope and coping data were collected at two time points: before and after program completion.

The Herth Hope Index (HHI) measures levels of hope [20] and is commonly used in clinical settings, research, and bereavement studies [21]. The HHI consists of 12 items, each rated on a 4-point scale (1 = strongly disagree, 2 = disagree, 3 = agree, 4 = strongly agree). Total hope scores can range from 12 to 48. The HHI includes 3 subscales: (1) temporality and future, (2) positive readiness and expectancy, (3) interconnectedness. According to the original authors, the scale has strong internal consistency with an alpha coefficient of 0.97 [20].

The Brief Cope Scale was used to measure coping levels [22,23]. This 28-item scale assesses adaptive (i.e., active coping) and maladaptive (i.e., substance use) coping strategies. Each item is rated on 4-point scale (1= I have not been doing this at all, 2 = a little bit, 3 = a medium amount, 4 = I have been doing this a lot). The items are divided into 14 coping strategies: (1) self-distraction, (2) active coping, (3) denial, (4) substance use, (5) emotional support, (6) instrumental support, (7) behavioral disengagement, (8) venting, (9) positive reframing, (10) planning, (11) humor, (12) acceptance, (13) religion and (14) self-blame. An internal consistency of over 0.50 was reported for each subscale, indicating adequate reliability [22].

#### 2.2.2. Semi-Structured Interviews

An interview guide was developed by the authors using Sekhon and colleagues’ framework on healthcare intervention acceptability [24] and consisted of seven open-ended questions (Table S2). Interviews were conducted either via the Zoom platform, by phone or in-person at the Jewish General Hospital in Montreal based on participants’ preferences and were audio-recorded. Each interview lasted between 30 and 60 min. Eight of the 11 participants were interviewed at three time points: before, during and after program completion. Three participants were interviewed only twice (at midpoint of program and after program completion).

### 2.3. Data Analysis

#### 2.3.1. Quantitative Data

The IBM SPSS Statistics software (Statistical Package for Social Science), version 29 was used [25] to compute descriptive statistics for socio-demographic characteristics and levels of hope and coping,. which were reported as means with standard deviations (SDs) as well as minimum and maximum values for continuous variables, and counts with percentages for categorical variables. If the quantitative data were normally distributed, paired-sample *t*-tests were used to compare pre- and post-test scores; otherwise, the Wilcoxon signed-rank test was used [26,27,28]. Although normality analysis suggested that 7 of the 14 coping variables were not normally distributed (Shapiro–Wilk test, *p* < 0.05), the Q-Q plots indicated normality of change scores. Given the robustness of paired-sample *t*-tests and the absence of clear outliers, paired-sample *t*-tests were used to compare coping levels before and after program attendance. A Wilcoxon signed-rank test of participants’ coping scores before and after program completion was also conducted, given the small sample size [28]. A significance level of 0.05 (two-tailed) was adopted for all statistical tests. Cohen’s d and r were reported as measures of effect size for the paired-sample *t*-tests and Wilcoxon signed-rank tests, respectively. Negatively keyed items for the HHI were reverse-scored. HHI scores were calculated using available item responses and no imputation was performed for missing data. Sum scores were then used to analyze the 3 HHI subscale scores and total hope (sum of all 3 subscales) scores before and after program completion. Two participants had missing item responses; however, subscale scores could still be calculated using available responses. Therefore, all pre- and post-program subscale scores were retained for analysis.

No reverse coding was used for the Brief Cope Scale (BCS), following the established scoring protocol [22]. Mean scores within each subscale (i.e., the total score for 2 items divided by 2) were calculated to make scores more interpretable based on the original 1- to 4-point scale. BCS scores were calculated using available item responses. Only participants with complete pre- and post- program cope subscale scores were included in the paired comparisons. A participant who did not complete any BCS items was therefore excluded from this analysis (*N* = 10). Participants with some missing pre- or post-program subscale scores were excluded from the analyses for that specific subscale. Subscales with missing items were treated as missing, and no imputation was performed. In addition, Cronbach alpha was not computed due to the small sample size [29]. Given the exploratory nature of this pilot study and the small sample size, corrections for multiple comparisons were not applied for the BCS.

#### 2.3.2. Qualitative Data

Interview data were analyzed using an inductive, thematic approach [30]. Audio-recorded content was transcribed verbatim. Two members of the research team (Y.K as the primary author and S.A., Saima Ahmed, Ph.D.) thoroughly and independently read the transcripts. Transcripts were coded by Y.K. and emerging themes were identified, reviewed and discussed jointly. Relevant statements were categorized into themes and subthemes. Disagreements were discussed until consensus was reached. The senior author (C.G.L) reviewed all resulting themes, subthemes and quotes.

All members of the research team have psychosocial oncology training. At the time of the study, Y.K. and S.A. were graduate students with relevant coursework and research experience. C.G.L. is a full professor with over 25 years of research and mentorship experience in psychosocial oncology. Initial contact with participants was established during the phone screening by Y.K., during which the rationale for and interest in the research topic were discussed. Given the team’s professional and academic backgrounds, this may have shaped how participant’ experiences were interpreted. However, familiarity with the subject matter facilitated building rapport and sensitivity during interviews. Reflexivity was supported through discussions throughout the process of data analysis (i.e., during coding and theme development). These provided opportunities for team members to reflect on how their perspectives and experiences may have influenced data interpretation.

## 3. Results

### 3.1. Participant Characteristics

The study sample (*N* = 11) included nine women and two men between 31 and 89 years of age. All participants reported not currently living with someone and not having any dependents. Eighty-two percent (*n* = 9) of the participants identified as White with a Canadian or European background. More than half (*n* = 6) had a university degree (undergraduate or graduate). A total of 64% (*n* = 7) were retired, and 73% (*n* = 8) reported having lost a spouse between two and 11 months prior to program registration (Table 1).

### 3.2. Quantitative Results

#### 3.2.1. Herth Hope Index

Table 2 summarizes the differences in participants’ levels of hope before and after program completion. The Shapiro–Wilk normality test revealed that the score differences were normally distributed (*p* > 0.05). Therefore, participants’ hope levels before and after program completion were compared using a paired-sample *t*-test. Thetotal hope score was significantly increased after program completion (M_difference_ = 3.1, t(10) = −3.078, *p* = 0.012, 95% CI [−5.328, −0.0854], d = 0.93). Within the three aspects of hope, a significant increase was observed in the temporality and future aspect (M_difference_ = 1.64, t(10) = −4.845, *p* < 0.001, 95% CI [−2.389, −0.884], d = 1.46). No significant differences were seen in the positive readiness and expectancy and interconnectedness subscales (*p* > 0.05).

#### 3.2.2. Brief Cope Scale

The differences in participant levels of cope pre- and post- are summarized in Table 3 and Table 4. No significant differences were observed in participant coping levels across the 14 subscales pre- and post-program completion using either paired sample *t*-tests (Table 3) or Wilcoxon signed-rank tests (Table 4) (*p* > 0.05).

### 3.3. Qualitative Findings

#### 3.3.1. Theme 1: Tangible and Intangible Program Contributions to Bereavement Processes

At T1, all participants entered the program with an open mindset. One participant described the program as an opportunity to move forward, explaining, *“I think it’s a stepping stone, you know? […] the door is open and it’s up to you to walk through. So this is one I’m going to walk through and I’ll see what happens when I get there.”* (female, age 73, widowed). Some participants (4/8) did not have any expectations about the program and were unsure whether they would benefit. One of these participants mentioned the value of speaking with the program facilitator in advance, noting that this interaction helped them gauge how the program would be conducted. She stated, *“The one thing that impressed me is what a great listener she [the facilitator] is. […] That in itself kind of constructed in my mind, what this program is going to lead to and the person that’s going to be leading it, cheering it, facilitating it. I’ve already formed an opinion about [the facilitator].”* (Female, age 64, widowed).

Many participants (5/8) hoped to gain new perspectives and regain a sense of purpose. Such comments included:

*“Maybe there’s ways that people deal with it that I can’t imagine […] my idea is hopefully to turn my head around and think of getting something out of it and not being cynical.”*

(Female, age 89, widowed)

*“Hopefully it [the program] can show us that there is life beyond this. That you can find a purpose, a meaning.”*

(Male, age 77, widowed)

Having registered to the program, participants also looked forward to having their own dedicated time and space to talk about their grief, gain coping skills, and receive informational support. One participant shared, *“Since he died, I never really had time to sit down. When I heard about this program, I said, ‘oh, I need an hour and a half just for me.’”* (Female, age 77, widowed).

By T2, some participants (5/11) remained unsure about the extent to which they were benefiting from the program, as one shared, *“I’m not sure yet. This will only be the 5th [session], so I don’t want to give a judgment.”* (Female, age 73, widowed). The remaining participants shared their perceived benefits from the program. These included intangible benefits such as shifting their emotions, gaining new perspectives on their situation, and making progress in processing their grief. For example, a participant reflected on how listening to the experiences of other attendees enhanced their understanding of the depth and diversity of grief beyond their own experiences. *“She lost her son to cancer. […] It kind of knocks you off your feet a little bit. It shows that the gravity of loss is not just husband and wife, et cetera. We kind of get wrapped up in our own little world.”* (Male, age 77, widowed).

Tangible program contributions were also identified, including the development of coping strategies, time to process their grief, and the ability to articulate one’sfeelings. Participants reflected on these aspects:

*“It’s been hopeful in terms of developing coping strategies or just even think about things differently.”*

(Male, age 31, lost parent)

*“It’s giving me time to think.”*

(Male, age 77, widowed)

*“I’m a little slower in processing my guilt and grief, but I’m getting there. At least I can talk about it.”*

(Female, age 62, lost parent)

At T3, most participants (8/11) felt the program was beneficial, as they described a broader range of ways the program had contributed to their bereavement processes. Similar to T2, tangible benefits included developing coping strategies and the ability to better articulate their grief. In addition to the intangible benefits already mentioned at T2, new perceived benefits emerged such as gaining a sense of identity, independence and strength.

*“I think that from now on, life will be different because I found who I am. Because when you’re married for 35 years, you tend to be one and sometimes you lose your identity. This time. I found my identity.”*

(Female, age 77, widowed)

*“I’m making on my own and going forward. I’ve been doing my banking. I’m checking things out. What’s coming in, what’s going out? It’s a different way of life, you know. But it is life […] Living, living that life. Keep trying.”*

(Female, age 73, widowed)

*“I think it’s kind of giving me a little boost along the way now. For awhile I was very weak, fragile and didn’t nearly know which way to turn, to not as weak, not as fragile. I still hurt inside but it has helped my progression through grief.”*

(Male, age 77, widowed)

These findings demonstrated a contrast with how participants had felt before starting the program, as they had previously mentioned feeling lost and no longer feeling like themselves:

*“I feel I don’t know what I want.”*

(Female, age 89, widowed)

*“I don’t cook for myself anymore.”*

(Female, age 64, widowed)

*“I don’t understand anything.”*

(Female, age 77, widowed)

At the program completion, two participants recommended a more active promotion of the program. *“Most of us had heard of Hope & Cope, but when you think about that, you think of Hope & Cope as they’re there as you’re going through your cancer treatment. But to be there afterwards—a program afterwards for grieving? I’m not the only one to have felt that. […] There has got to be some advertisement because we felt that the program really does help,”* said one participant. (Female, age 64, widowed). While many felt they had made meaningful progress, several (5/11) also acknowledged that their bereavement journey was ongoing, and that further support would be beneficial.

#### 3.3.2. Theme 2: Enhanced Sense of Community Carried Post-Attendance

At T1, many participants (5/8) mentioned feeling lonely and wondered if they were alone in how they were feeling. Most (6/8) felt that they were not fully understood by friends or family members because *“like anything in life, if you don’t experience it, you truly don’t know what it’s like.”* (Female, age 64, widowed). Knowing they were going into the program with others with shared losses, many mentioned they saw the group members as “equals”.

*“We’re all starting the same […] we’re all going through the same thing.”*

(Female, age 73, widowed)

*“When we go in there, we’re basically all going in there equal. That’s how I look at it.”*

(Male, age 77, widowed)

Two participants wondered if they would be able to share with this group due to their shy personalities. One participant wondered if she *“will have the patience to hear all kinds of stories.”* (Female, age 89, widowed)

At T2, most (10/11) participants expressed a positive outlook toward the group, mentioning they felt at ease expressing themselves. The group was described as a safe space where participants could be themselves and not be judged for how they felt due to the shared experience of grief. As one participant shared, *“It’s allowed me to open up and not be afraid to be me, not be judged.”* (Female, age 65, widowed).

A participant described how shared experiences of cancer-related loss within the group created a space to discuss complex family dynamics surrounding grief. “*People were talking about different situations and then I related when my mother passed away. She had cancer. […] [Her passing] caused so much havoc to this day. [My siblings and I] used to be close. So I had said to the group that I lost my mother, but I lost my siblings as well. So I had all that baggage besides losing my husband.”* (Female, age 73, widowed)

Others reflected on the sense of connection that emerged from shared bereavement experiences:

*“You feel at ease because the people that you’re relaying your story to are exactly at the same place that you are. The understanding is there, whereas when you’re talking to family or you’re talking to friends, you don’t.”*

(Female, age 64, widowed)

*“It’s amazing how you can put people in a room who are going through the same process of grief to a point, how easily those 14 people, who could walk down the street and walk by each other and never say hello, […] are able to equate and talk back and forth to each other with ease.”*

(Male, age 77, widowed)

Many participants (8/11) shared that being part of the group helped normalize their feelings. As one stated, *“it made me feel not so abnormal, that other people are having difficulties dealing with grief.”* (Female, age 62, lost parent). One participant looked forward to growing alongside others in the group: *“We go in there as 11 different units, but we walk out of there feeling for each other […] I’d like to see how we, all of us, evolve out of it to a point from where we were when we went, when we were 11 fractured people as to where we are then.”* (Male, age 77, widowed).

This concept of group growth was mentioned by four more participants at T3. One participant noted, *“you see the difference from the first week to where we were this past week, like people brought in baked goods and shared memories and stuff like that.”* (Male, age 31, lost parent).

At the end of the program, most participants (9/11) reported having made meaningful connections and expressed a desire to stay in touch beyond the program. As the program was ending, participants mentioned that they were given options to join other support groups offered by Hope & Cope. However, they were hesitant to separate from the existing group, emphasizing that *“it takes time to create that bond”* (female, age 65, widowed) and preferred to keep the connections they already made and follow up with each other. Others shared similar sentiments:

*“I made some connections. I’m going to continue seeing those people.”*

(Female, age 83, widowed)

*“The group decided that it would be nice just to check up on everyone.”*

(Male, age 31, lost parent)

*“[Program facilitator] has also mentioned the additional programs—the Mourning Walk and the Mourning Café […] and we can also join with another group. But we got close to our little… it’s referred as “participants” but I think most of the participants now see it as our little grieving family.”*

(Female, age 64, widowed)

Conversely, one participant notably did not share the same sense of connection. While the majority of participants (8/11) had lost a spouse, this participant had experienced a different type of loss and felt out of place within the group. They expressed feeling unable to relate and found it difficult to share their story with the group:

*“I kept thinking all the time that I’m different from this group. It’s not the same losing your son, and losing a sibling or parent or a husband. I’m not at all in the same situation. I couldn’t even talk about my situation really. […] It added to the weight on my shoulders to hear others talk about their loss, which I thought could not be compared to me, so it made me feel worse.”*

(Female, age 83, lost child)

#### 3.3.3. Theme 3: Preference for In-Person vs. Virtual Delivery

Before the program began (T1), most participants (7/8) expressed a preference for in-person program delivery. They valued the connections that could be made through in-person, sharing that *“over the phone, or over the internet, zoom, […] it becomes very impersonal,”* (female, age 64, widowed) and feels *“awkward to sit in front of a computer to talk about anything and just seeing a face.”* (Female, age 83, lost child). However, with the program delivered in-person, some participants (4/8) anticipated challenges associated with attending in-person sessions, including scheduling conflicts, parking, and fear of showing to show their emotions. These comments included:

*“I’m already thinking, ‘where am I going to park?’”*

(Female, age 64, widowed)

*“I’m afraid I’m going to cry.”*

(Female, age 73, widowed)

*“I just pray I don’t get called in from my boss on Tuesdays because I really want to go to these meetings.”*

(Female, age 62, lost parent)

At T2, participants’ preference for in-person sessions remained consistent, although practical barriers such as scheduling conflicts and parking continued to cause stress. One participant shared, *“I have to run through traffic […] and then to try to park. So it’s a little bit stressful for me.”* (Female, age 65, widowed).

Still, two participants mentioned that the program served as a motivator to go outside, with one participant stating, *“I know I have some place to go, it just gets me out of the house.”* (Female, age 73, widowed). Participants also highlighted that being physically present allowed them to feel heard, seen and more engaged during sessions:

*“When they see that you’re not having a great moment, the pat on the back or the hug, you know? Or the Kleenex.”*

(Female, age 73, widowed)

*“You could tell by a person’s behavior, even facial expressions, how they feel. I need that feedback also. I need that assurance […] it’s important, instead of saying on the phone. It’s not as personal, whereas in a meeting, it’s very personal.”*

(Female, age 62, lost parent)

For one, this in-person experience also enhanced the retention and practical application of what participants had learned when facing difficult moments:

*“I like to be in-person because […] when I walk out of that room, I know exactly where everybody was seated, and I remember what they say. I remember actions, I remember and that helps me.”*

(Female, age 64, widowed)

At T3, participants mentioned that in-person delivery not only helped them to connect, but also confronted the absence of physical touch they used to have with their loved ones as *“part of missing them immensely is missing being able to hold their hand, being able to give them a hug, give them a kiss, tell them ‘I love you.’”* (Male, age 77, widowed). Participants also valued the informal conversations that occur before and after in-person sessions, which helped foster connections and thus highlighted them as a limitation of virtual program delivery: *“I don’t get to talk to you before the class or the before the session. I don’t get to talk to you afterwards. You know? It’s just not the same.”* (Female, age 64, widowed).

While the overall sentiment favored in-person delivery, a participant who had previously attended a virtual support program, acknowledged the benefits of virtual options, including the use of displayed names and the convenience of attending the program: *“It was easier for me to get home. You know, do what I have to do and then sit down and be at the 5:30 [meeting] […] and having name tags for the people that are there as a group.”* (Female, age 65, widowed).

Ultimately, all participants shared that they liked the program being delivered in-person, emphasizing the in-person connections and the sense of place it provided:

*“I’m very appreciative of the fact that it was in-person, a place to be.”*

(Female, age 77, widowed)

*“I still believe I would not have that connection if we were doing this online. […] You give me a choice to do something zoom or do it in person, I would choose in-person all the time. It comes with more positives […] being in person and meeting these people. I feel that I formed a connection with them and they formed a connection with me.”*

(Female, age 64, widowed)

## 4. Discussion

This pilot study sought to assess the impact of an eight-session community-based bereavement support program on hope, coping, and bereavement-related experiences. Hope significantly increased post-program. A significant increase was also observed in the “temporality and future” aspect of hope. According to Herth [20], this suggests that perceiving more positive future outcomes is significantly linked to higher hope. This observation was further supported by qualitative interviews in which participants expressed a more positive outlook on moving forward with their grief at program completion. These findings are consistent with evidence elsewhere demonstrating the positive effects of peer support programs on hope [31,32,33]. Interestingly, the HHI subscale “interconnectedness”—which assesses individuals’ sense of interpersonal connections [20]—was not significantly correlated with program participation, although interviews revealed that participants experienced an enhanced sense of community by the program’s end. This discrepancy may be attributable to the limited sample size and warrants further investigation with a larger sample. Given the significant relationship between hope and the bereavement program, hope may be a promising construct to explicitly include in future cancer support program assessments.

Participant cope scores were not statistically significant pre- and post-program completion. This may be due, in part, to the fact that participants had not yet applied learned coping skills in their daily lives. Second, several participants expressed that additional sessions would have been appreciated. Indeed, eight sessions may not have been long enough to capture significant changes in coping, given that behavioral changes take time [34,35]. Thus, investigating the longer-term impact on coping may be worth pursuing. Although changes in coping levels were not statistically significant at program completion, moderate effect sizes (Cohen’s d > 0.5) were seen in several domains such as “use of informational support” and “positive reframing”. This may suggest that the program has the potential to change participant coping levels in these domains. The absence of significance may also primarily reflect a Type II error associated with limited statistical power given the small sample size. It would be worth replicating the study with (1) a larger sample size to determine if variables of moderate effect sizes become statistically significant, and (2) a control group to determine if changes in coping can be directly attributed to program attendance.

The qualitative data revealed several program benefits including gaining new perspectives, developing an enhanced sense of self, and normalizing feelings—these are consistent with prior research [36,37]. Interestingly, participants rarely discussed losing a loved one specifically to cancer. This may be due to a focus on their bereavement processes rather than the cause (i.e., the illness) that contributed to their grief. Previous studies show that bereavement groups can generate a sense of coherence and provide a safe environment for participants to express their feelings [38,39]. Similarly, our findings indicate that participants experienced an enhanced sense of community characterized by shared experiences, emotional safety, and perceptions of collective growth. Of note, one participant reported feeling that they did not have a sense of group belonging, having lost a child rather than a spouse. This aligns with existing research underscoring that bringing together participants with similar losses contributes to more positive outcomes [36].

In sociodemographic terms, more than half of the participants were over 70, were retired and all lived alone. Previous research suggests that factors associated with aging such as reduced social interactions, widowhood and limited financial resources can reduce hope [40,41,42]. Hope,—a strong predictor of better physical and mental well-being—has been shown to be modifiable in older adults [43,44], which is consistent with our observations. As such, hope can act as an important resource for older adults navigating adversity and difficult life transitions such as bereavement. Because all participants were living alone during group attendance, they may have faced an increased vulnerability to stressors, particularly as social isolation and loneliness are associated with adverse health outcomes including depression [45,46]. Within this context, reduced daily support may significantly shape coping throughout bereavement and support group interactions may address this by providing opportunities to share and listen to each other’s experiences.

Whereas several studies have explored the benefits and potential drawbacks of in-person and virtual program delivery, our findings align with prior research highlighting the key advantages of in-person formats. According to participants, in-person meetings facilitated the processing of emotions and allowed for non-verbal expressions of support (i.e., handing someone a tissue when crying). These elements have previously been identified as limitations in virtual settings [47,48]. Participants also appreciated the in-person format as a motivator to leave their homes. As the sample herein included only people living alone, future research could explore if in-person preferences extend to individuals living with others.

As mentioned previously, there are a few limitations to this study. Participants voluntarily agreed to take part in the study, and self-selection bias may have been operative. Because data collection relied on participant self-report, no objective assessments of the program’s effects were conducted. Due to the small sample size and the lack of a control group, we cannot ascertain whether changes in hoping/coping levels were directly linked to program attendance or other factors such as simple social interactions and the Hawthorne effect. Given that the majority of the study sample were women who had experienced spousal loss, the qualitative findings may not be transferable across diverse populations with differing bereavement relationships. Finally, the 14 Brief Cope subscales were examined without corrections for multiple comparisons; therefore, the probability of a Type I error was increased.

## 5. Conclusions

This pilot study provides preliminary insights into the impact of an 8-session community-based support program for bereaved individuals. The program was associated with increased hope at program completion. In-depth interviews suggested that the program was perceived to be relevant and beneficial for regaining a sense of self, normalizing grief experiences, and enhancing a sense of community. In addition, gauging participants’ experiences with the program over time offers opportunities to adjust the program’s content and features as it unfolds.

## Figures and Tables

**Table 1:** Participant characteristics (*N* = 11).

Participant Characteristics	*N*	%	Mean (SD)	Range
**Biological sex**				
Female	9	81.8		
Male	2	18.2		
**Age (years)**			70.64 (15.67)	31–89
30–39	1	9.1		
40–49	0	0		
50–59	0	0		
60–69	3	27.3		
70–79	4	36.3		
80–89	3	27.3		
**Marital status**				
Widowed	9	81.8		
Single (or never married)	2	18.2		
**Currently living with someone**				
Yes	0	0		
No	11	100		
**Dependents**				
None	11	100		
**Ethnicity**				
White (Canadian/European)	9	81.8		
Latin American	1	9.1		
South East Asian	1	9.1		
**Level of Education Completed**				
University (undergraduate)	4	36.3		
University (graduate: master’s, doctorate or post-doctorate)	2	18.2		
High school	3	27.3		
Technical or vocational school or pre- university degree	2	18.2		
**Work status**				
Retired	7	63.6		
Full time (>30 h/week)	1	9.1		
Part time (<30 h/week)	0	0		
Self-employed	2	18.2		
Disability/sick leave	1	9.1		
**Relationship to Deceased**				
Spouse	8	72.7		
Parent	1	9.1		
Child	2	18.2		
A mount of Time since Passing of Loved One (months) *			6.1	2–11
0–6	6	54.6		
7–12	4	36.4		

* Adds up to less than 100% due to non-responses.

**Table 2:** Paired-sample *t*-test of participant hope scores before and after program completion (*N* = 11).

	Pre-Program Mean Sum Score (SD)	Post-Program Mean Sum Score (SD)	*p*-Value	*t*-Statistic (df)	Cohen’s d
Hope total	29.45 (10.23)	32.55 (9.41)	0.012 *	−3.078 (10)	0.93
Hope—temporality and future	9.18 (3.66)	10.82 (3.57)	<0.001 **	−4.845 (10)	1.46
Hope—positive readiness and expectancy	10.73 (3.04)	11.64 (2.42)	0.064	−2.085 (10)	0.63
Hope—interconnectedness	9.55 (3.91)	10.10 (3.94)	0.216	−1.322 (10)	0.40

Two participants had some missing values on the Herth Hope Index, however all pre-post subscale scores could still be calculated; * *p* < 0.05, ** *p* < 0.01.

**Table 3:** Paired-sample *t*-test of participant cope scores before and after program completion (*N* = 10; due to 1 non-response).

	Pre-Program Mean Sum Score (SD)	Post-Program Mean Sum Score (SD)	*p*-Value	*t*-Statistic (df)	Cohen’s d
1. Active coping (2 items)	2.55 (0.83)	2.75 (0.75)	0.269	−1.177 (9)	0.372
2. Use of informational support (2 items)	2.55 (0.64)	2.85 (0.53)	0.111	−1.765 (9)	0.558
3. Positive framing (2 items)	2.00 (1.03)	2.33 (1.00)	0.169	−1.512 (8)	0.504
4. Planning (2 items)	2.75 (0.98)	2.75 (0.68)	1.000	0.000 (9)	0.000
5. Emotional support (2 items)	2.80 (0.63)	2.95 (0.69)	0.560	−0.605 (9)	0.191
6. Venting (2 items)	2.33 (0.75)	2.17 (0.66)	0.397	0.894 (8)	0.298
7. Humor (2 items)	1.25 (0.63)	1.30 (0.63)	0.678	−0.429 (9)	0.136
8. Acceptance (2 items)	2.65 (1.13)	2.70 (0.89)	0.811	−0.246 (9)	0.078
9. Religion (2 items)	2.00 (1.08)	2.25 (0.98)	0.138	−1.627 (9)	0.514
10. Self-blame (2 items)	1.85 (0.85)	2.00 (0.85)	0.647	−0.474 (9)	0.150
11. Self-distractions (2 items)	2.95 (0.64)	3.05 (0.60)	0.642	−0.480 (9)	0.152
12. Denial (2 items)	2.30 (1.18)	2.05 (1.04)	0.299	1.103 (9)	0.349
13. Substance use (2 items)	1.35 (0.53)	1.20 (0.42)	0.520	0.669 (9)	0.212
14. Behavioral disengagement (2 items)	1.61 (0.74)	1.17 (0.25)	0.121	1.735 (8)	0.578

4 participants had some missing values in the Brief Cope Scale.

**Table 4:** Wilcoxon signed-rank test for participant cope scores before and after program completion (*N* = 10; due to 1 non-response).

	Pre-Program Mean Sum Score (SD)	Post-Program Mean Sum Score (SD)	*p*-Value	Z	r
1. Active coping (2 items)	2.55 (0.83)	2.75 (0.75)	0.279	−1.081 ^b^	0.44
2. Use of informational support (2 items)	2.55 (0.64)	2.85 (0.53)	0.109	−1.604 ^b^	0.93
3. Positive framing (2 items)	2.00 (1.03)	2.33 (1.00)	0.102	−1.633 ^b^	0.94
4. Planning (2 items)	2.75 (0.98)	2.75 (0.68)	0.666	−0.431 ^a^	0.16
5. Emotional support (2 items)	2.80 (0.63)	2.95 (0.69)	0.680	−0.412 ^b^	0.18
6. Venting (2 items)	2.33 (0.75)	2.17 (0.66)	0.366	−0.905 ^a^	0.32
7. Humor (2 items)	1.25 (0.63)	1.30 (0.63)	0.655	−0.447 ^b^	0.32
8. Acceptance (2 items)	2.65 (1.13)	2.70 (0.89)	1.000	0.000	0
9. Religion (2 items)	2.00 (1.08)	2.25 (0.98)	0.129	−1.518 ^b^	0.68
10. Self-blame (2 items)	1.85 (0.85)	2.00 (0.85)	0.863	−0.172 ^b^	0.06
11. Self-distractions (2 items)	2.95 (0.64)	3.05 (0.60)	0.603	−0.520 ^b^	0.20
12. Denial (2 items)	2.30 (1.18)	2.05 (1.04)	0.285	−1.069 ^a^	0.62
13. Substance use (2 items)	1.35 (0.53)	1.20 (0.42)	0.496	−0.680 ^a^	0.30
14. Behavioral disengagement (2 items)	1.61 (0.74)	1.17 (0.25)	0.102	−1.633 ^a^	0.73

Four participants had some missing values in the Brief Cope Scale; Test statistic (Z), Effect size (r); ^a^ Based on positive ranks, ^b^ Based on negative ranks.

## Data Availability

Data from this study are available from the authors upon request.

## Supplementary Materials

The following supporting information can be downloaded at: https://www.mdpi.com/article/10.3390/curroncol33060350/s1, Table S1: *Living with Loss* program’s general topics; Table S2: Semi-structured interview guide.

## Abbreviations

The following abbreviations are used in this manuscript:

HHI	Herth Hope Index
M_difference_	Mean difference between pre- and post-program scores (Post–Pre)
