# Supplementary material for: Participants’ Evolving Experiences, Hope, and Coping While Enrolled in a Community-Based Bereavement Support Program: A Pre–Post Mixed-Methods Pilot Study
Source: Curr Oncol. 2026 Jun 10;33(6):350. doi: 10.3390/curroncol33060350 (PMC13297984; doi:10.3390/curroncol33060350)
Supplement: Supplementary file 1 [file curroncol-33-00350-s001.zip › Table S1. Living with Loss program's general topics.pdf]

Article: **Participants’ Evolving Experiences, Hope, and Coping While Enrolled in a Community-Based Bereavement Support Program: A Pre–Post Mixed-Methods Pilot Study**

Supplementary Material

Table S1. *Living with Loss* program’s general topics.

LIVING WITH LOSS	**TOPICS**
**SESSION 1**	Welcome – Guidelines for the group – sharing stories
**SESSION 2**	Symptoms of grief and typical reactions
**SESSION 3**	Feelings and emotions associated with grief
**SESSION 4**	Coping and support
**SESSION 5**	Spirituality and significant days
**SESSION 6**	Memories: Sharing photos and keepsakes
**SESSION 7**	Grief and music
**SESSION 8**	Finding Meaning, connection, acceptance – Introspection/Where do I go now?
