# Supplementary material for: Participants’ Evolving Experiences, Hope, and Coping While Enrolled in a Community-Based Bereavement Support Program: A Pre–Post Mixed-Methods Pilot Study
Source: Curr Oncol. 2026 Jun 10;33(6):350. doi: 10.3390/curroncol33060350 (PMC13297984; doi:10.3390/curroncol33060350)
Supplement: Supplementary file 1 [file curroncol-33-00350-s001.zip › Table S2. Semi-structured interview guide.pdf]

Article: **Participants' Evolving Experiences, Hope, and Coping While Enrolled in a Community-Based Bereavement Support Program: A Pre–Post Mixed-Methods Pilot Study**

Supplementary Material

Table S2. Semi-structured interview guide.

*Brief script:*

*Thank you for taking the time to talk to us about your perceptions or expectations of the "Name of the program" you are currently enrolled in at Hope & Cope.*

*We would like to ask you a few questions about the Hope & Cope program you are enrolled in. that you will begin attending (before program attendance),OR your experience with the program so far (during program attendance),OR your overall experience with the program you were enrolled in (after program completion). Please feel free to share with us any thoughts. There are no right or wrong answers.*

*If you feel distressed or any negative emotions at any point during the interview, please let me know and we can stop the interview. I can have the Hope & Cope program coordinator contact you for support, if you wish.*

| | *Before program* | *During program* | *After program* |
|---|---|---|---|
| **1) Affective Attitude Item** *how an individual feels about taking part in a program* | Is there anything that *you expect to like* about (The Program)? | Is there anything that *you like, so far,* about (The Program)? | Is there anything that *you liked* about (The Program)? |
| 2) **Burden Item** *perceived amount of effort that is required to participate in the program* | How easy or difficult *will it be* to participate in (The Program)? | How easy or difficult *has it been* to participate in (The Program)? | How easy or difficult *was it* to participate in (The Program)? |
| **3) Effectiveness Item** *the extent to which the program is perceived* | To what extent do you think (The Program) *will make* a difference in the lives | To what extent do you think (The Program) *is making* a difference in the lives of people attending it? | To what extent do you think (The Program) *made* a difference in the lives of people attending it? |

| | | | |
|---|---|---|---|
| *to make a difference in participants* | of people attending it? | | |
| **4) Opportunity Costs Item** *the extent to which benefits, profits, or values must be given up to engage in a program* | Is there anything in particular that you personally *will be giving up or sacrificing* to participate in (The Program)? | Is there anything in particular that you personally *are giving up or scarifying* to participate in (The Program)? | Is there anything in particular that you personally *had to give up or sacrificed* to participate in (The Program)? |
| **5) Intervention Coherence Item** *the extent to which the participant understands the program, and how it works* | Do you know the objectives of the program *you will attend*? | Do you have a better understanding of the objectives of the program, now *after attending few sessions*? | Do you have a clear understanding of the objectives of the program *you were enrolled in*? |
| **6) Self-Efficacy Item** *participant confidence that they can perform the behavior(s) required to participate in the program* | In your opinion, how confident are you that you *will be able* to perform the tasks required by (The Program)? | In your opinion, how confident are you that you *are able* to perform the tasks required by (The Program)? | In your opinion, how confident are you that you *were able* to perform the tasks required by (The Program)? |
| **7) Ethicality Item** *the extent to which the intervention has good fit with an individual's value system* | To what extent do you think (The Program) *will be* a good fit with your own values/beliefs? | To what extent do you think (The Program) *is* a good fit with your own values/ beliefs? | To what extent do you think (The Program) *was* a good fit with you own values/ beliefs? |

*Would you like to share any additional thoughts or suggestions about the program?*
